# Association between Salivary Cortisol and α-Amylase with the Psychological Profile of Patients with Oral Lichen Planus and Burning Mouth Syndrome: A Case–Control Study

**DOI:** 10.3390/biomedicines11082182

**Published:** 2023-08-03

**Authors:** Ana Glavina, Liborija Lugović-Mihić, Dinko Martinović, Livia Cigić, Leida Tandara, Marino Lukenda, Dolores Biočina-Lukenda, Daniela Šupe-Domić

**Affiliations:** 1 Dental Clinic Split, 21000 Split, Croatia; glavina2014@gmail.com (A.G.); dbiocina.lukenda@gmail.com (D.B.-L.); 2 Department of Oral Medicine and Periodontology, Study of Dental Medicine, School of Medicine, University of Split, 21000 Split, Croatia; livia.cigic@mefst.hr; 3 Department of Dermatovenereology, University Hospital Center Sestre Milosrdnice, 10000 Zagreb, Croatia; liborija@gmail.com; 4 School of Dental Medicine, University of Zagreb, 10000 Zagreb, Croatia; 5 Department of Maxillofacial Surgery, University Hospital of Split, 21000 Split, Croatia; d.m.993@hotmail.com; 6 Department of Pathophysiology, School of Medicine, University of Split, 21000 Split, Croatia; 7 Department of Dental Medicine, University Hospital of Split, 21000 Split, Croatia; 8 Department of Medical Laboratory Diagnostics, University Hospital of Split, 21000 Split, Croatia; leida.tandara@gmail.com; 9 Study in English, School of Medicine, University of Split, 21000 Split, Croatia; marino.lukendaa@gmail.com; 10 Department of Health Studies, University of Split, 21000 Split, Croatia

**Keywords:** alpha-amylase, biomarkers, burning mouth syndrome, cortisol, oral lichen planus, saliva

## Abstract

The aim of our study was to assess the relationship between the concentration/activity of salivary stress biomarkers (cortisol, α-amylase) and the psychological profile of patients with oral lichen planus (OLP) and primary burning mouth syndrome (BMS). A total of 160 subjects participated in this case–control study: 60 patients with OLP; 60 patients with primary BMS; and 40 control subjects. Unstimulated whole saliva (UWS) was collected between 9 and 10 a.m. Salivary biomarkers were analyzed by enzyme-linked immunosorbent assays (ELISAs). Psychological assessment was evaluated with the Depression, Anxiety, and Stress Scale (DASS-21). The patients with primary BMS had higher salivary cortisol concentrations and α-amylase activity (0.52 vs. 0.44 µg/dL; 160,531 vs. 145,804 U/L; one-way analysis of variance (ANOVA) with *post hoc* Scheffe test) compared with patients with OLP. The patients with primary BMS had statistically significant higher scores for depression, anxiety, and stress compared with patients with OLP and control subjects (*p* < 0.001, Kruskal–Wallis test). There was a strong positive correlation between anxiety and depression, stress and depression, and stress and anxiety in patients with OLP and BMS (*p* < 0.001 and *p* < 0.001, respectively; Spearman’s correlation). There was a good positive correlation between symptom intensity (pain/burning) and psychological profile (depression, anxiety, stress) in patients with primary BMS (r = 0.373, *p* = 0.003; r = 0.515, *p* < 0.001; r = 0.365, *p* = 0.004, respectively; Spearman’s correlation). This case–control study is the first to compare the psychoendocrinological profile of patients with two different oral diseases. The patients with BMS showed a higher concentration/activity of salivary stress biomarkers (cortisol, α-amylase) and a stronger association with mental disorders compared with patients with OLP. However, an interdisciplinary psychoneuroimmunological approach is equally important in both patient groups (OLP and BMS), regardless of whether mental disorders are the cause or the consequence.

## 1. Introduction

Oral lichen planus (OLP) is considered a chronic inflammatory autoimmune disease of unknown etiology that has phases of remission and relapse. There is a genetic predisposition for the occurrence of this disease and specific human leukocyte antigens (HLAs): A3, A8, B5, B7, B8. A number of possible triggers and contributing factors for OLP have been proposed: (1) local and systemic triggers of cell-mediated hypersensitivity; (2) stress; (3) autoimmune response to epithelial antigens; and (4) microorganisms [1]. The role of psychological stress is not clear, nor is the causal relationship between stress and the occurrence of OLP [2,3,4].

Burning mouth syndrome (BMS) is a complex chronic pain disorder characterized by spontaneous unpleasant sensations (burning, pain) of the clinically healthy oral mucosa, excluding all local and systemic causes [5]. The etiology is multifactorial and possible etiologic factors include local, systemic, psychological, neurological, and idiopathic factors [6]. Psychological disorders are more frequently described in patients with BMS and may trigger the onset of the disease, although studies have not confirmed the cause–effect relationship [7,8,9,10,11]. It remains unclear whether psychological factors underlie BMS or are its consequence.

The measured concentration of salivary cortisol is an indicator of the level of free cortisol or biologically active cortisol in human serum [12]. It remains an open question whether the hypothalamic–pituitary–adrenal (HPA) axis is hyperactive or hypoactive in response to acute stress when a person is exposed to chronic stress. Studies show that regulation of the HPA axis is impaired in the context of psychopathology, as evidenced by decreased fluctuations in salivary cortisol production [13]. In addition to cortisol, the central nervous system (CNS), which produces catecholamines, epinephrine, and norepinephrine, also plays an important role in stressful situations. The CNS regulates the activity of salivary α-amylase [14]. There are few studies on the relationship between chronic psychological stress (e.g., depression) and salivary amylase levels. The results suggest that salivary amylase may be a useful indicator of physiological changes in long-term exposure to stressful circumstances [15].

Psychoneuroimmunology is an interdisciplinary field that represents the convergence of psychology, neuroscience, endocrinology, and immunology. Various stressful stimuli perceived by the brain can trigger neurological activity, which then directly affects immune and endocrine responses. Psychological factors can cause stress-induced immunosuppression and inflammation, as well as various subtle changes in the regulation of the endocrine and immune systems that can alter the course of various diseases. Various oral diseases and conditions with a multifactorial etiology (periodontitis, OLP, recurrent aphthous stomatitis (RAS), temporomandibular disorders (TMD), herpes labialis, atypical odontalgia) may be influenced by psychoneuroimmunological interactions. Clinicians should be aware of the interplay between mental and general health in daily clinical work and consider treating mental disorders as an adjunct to conventional treatment modalities [16]. Therefore, it is important to develop noninvasive objective diagnostic tools to quantify mental disorders that would be useful in daily clinical work, such as salivary biomarkers.

The objectives of our study were to determine the concentration/activity of salivary stress biomarkers (cortisol, α-amylase), the psychological profile of patients with OLP and primary BMS, and the influence of disease duration and symptom intensity (pain/burning) on their salivary concentration/activity. Our case–control study is the first to compare the psychoendocrinological profile of patients with OLP and BMS (two oral diseases with different etiopathogenetic mechanisms).

The hypothesis of our study was that a higher concentration/activity of salivary stress biomarkers (cortisol, α-amylase) correlates with poorer mental health (higher levels of depression, anxiety, stress) in patients with OLP and BMS.

## 2. Materials and Methods

### 2.1. Study Design and Subjects

A total of 160 subjects, divided into three groups, participated in this case–control study: 60 patients with a clinically and histopathologically confirmed diagnosis of OLP; 60 patients diagnosed with primary BMS; and 40 control subjects. The group of patients with OLP was composed of 40 patients with an erosive form of the disease and 20 patients with a non-erosive form. The control group consisted of randomly selected patients who came to the Department of Diagnostic Radiology, Dental Clinic Split, Split, Croatia. The study protocol was explained to each subject and after signing the informed consent form, they were enrolled in the study. The study lasted two years (2020. to 2022.) and was conducted with the approval of the Ethics Committee of the Dental Clinic Split, the teaching base of the School of Medicine, Study of Dental Medicine, University of Split, Split, Croatia (approved on 24 July 2020) and the School of Dental Medicine, University of Zagreb, Zagreb, Croatia (05-PA-30-XIX-9/2020) (approved on 10 September 2020). The study was conducted in accordance with the principles of the Declaration of Helsinki (1964) and its subsequent amendments.

Medical history data, a list of medications, and disease duration (months) were obtained from all subjects. The same oral medicine specialist performed a clinical oral examination of all subjects and an incisional biopsy of the oral cavity mucosa in the patients with OLP. Local and systemic factors were removed in patients with primary BMS by determining whole saliva (sialometry test), complete blood count (CBC), serum iron (Fe), folic acid, vitamin B12, and blood glucose levels [17]. Blood pressure was measured in all subjects before participation in the study to exclude the possible influence of hypertension on the HPA axis and autonomic nervous system (ANS). Patients with inflammatory oral diseases (gingivitis, periodontitis) were also excluded from the study. All subjects who did not understand the nature and purpose of the study and the content of the informed consent form were excluded from the study.

The inclusion criterion for OLP was:

1. Patients with a clinically and histopathologically confirmed diagnosis of OLP according to the modified WHO criteria [18].

The exclusion criteria for OLP were:

1. Patients with long-term systemic diseases [*diabetes mellitus* (DM), cardiovascular disease (CVD), renal dysfunction, liver disease] and/or autoimmune diseases and/or cancer; pregnant women;
2. Patients who have received corticosteroid, immunosuppressive, or psychoactive therapy (anxiolytics, anticonvulsants, antidepressants) within the last three months or received hormone therapy;
3. Patients with harmful habits such as betel nut or tobacco chewing or smoking;
4. Cutaneous lichen planus (LP).

The five main inclusion criteria for primary BMS according to Scala A et al. [8] were:

1. A diffuse and usually bilateral burning sensation in the oral mucosa;
2. A constant and usually bilateral burning sensation that worsens during the day;
3. A burning sensation in the oral mucosa that has persisted for at least four to six months;
4. A burning sensation that does not interfere with the patient’s sleep;
5. A burning sensation that does not worsen with eating and drinking, and possible relief of discomfort with eating and drinking.

The exclusion criteria for primary BMS were:

- Fe, folic acid, vitamin B12 deficiency;
- DM;
- Receiving treatment with antihypertensive drugs (ACE inhibitors), drugs with xerostomic effects, drugs that make the oral cavity susceptible to the development of oral candidiasis (corticosteroids, antibiotics); taking antineoplastic, psychoactive, or neurological therapies in the last three months; or receiving hormone therapy;
- Smoker;
- Have oral mucosal disease (candidiasis); head and neck cancer; thyroid, liver, or kidney disease; allergies; gastroesophageal reflux disease (GERD); Sjögren’s disease (SjD);
- Pregnant or breastfeeding women;
- Underwent head and neck radiation.

### 2.2. Sample Size

The sample size was calculated using the statistical program G*Power to draw conclusions with sufficient statistical power [19]. An a priori power analysis was performed using a three independent subject group design, with the main interest on main fixed effects. Statistical power (1 − β) was set at 0.95 (α = 0.05; one-way test), whereas effect power was set at f = 0.30, i.e., the mean effect level considering the results of previous similar studies. The power analysis calculated that a minimum sample size of 177 subjects was required, which, in the context of this study and the specific interests of the researchers, would mean that the final sample at the level of a group should include approximately 60 subjects per group if the groups are of equal size. The same statistical program also calculated the required sample size to draw statistical conclusions with sufficient statistical power in the case of correlation plots across the entire sample. An a priori power analysis was performed, in which a linear regression design was chosen to test correlations. The statistical power (1 − β) is set at 0.95 (α = 0.05; two-way test), while the magnitude of the direction (eng. slope) or level of correlation at H1 is set at r = 0.3, i.e., at the small- to medium-effect level considering the results of previous studies. A required total sample size of 134 subjects was obtained by the analysis. The sample size in this study was determined by a more sophisticated design considering the nature of the hypotheses. Therefore, the planned number of subjects in the study was set at about 60 subjects per group, i.e., it should include about 180 subjects in total.

### 2.3. Saliva Sampling

Three days before saliva collection, all subjects were asked to refrain from intense physical activity and mental stress. All subjects were asked to refrain from eating, drinking, and brushing their teeth 90 min before sampling. Unstimulated whole saliva (UWS) was collected between 9 and 10 a.m. to avoid diurnal variation. The systematic review and meta-analysis by Fernández-Agra M et al. showed that studies in which both types of whole saliva [UWS and stimulated whole (SWS)] were collected yielded similar results in salivary biomarkers. They concluded that UWS is suitable for salivary biomarker detection and can serve as a reference for future studies [20]. UWS was collected from fertile women during the follicular phase of the menstrual cycle. Subjects were instructed to sit comfortably and tilt their head slightly forward. Immediately before collecting the saliva sample, all subjects rinsed their oral cavity with water to avoid contamination from other sources and then waited 10 min for the sample to be collected. They were instructed to swallow the saliva just before sampling began. Approximately 2.00 to 2.50 mL of saliva was collected from the subjects with OLP and BMS and the control subjects in graduated tubes (Salivette) (ref. 51. 1534.500, SARSTEDT AG & Co. KG, Nümbrecht, Germany) using the “spit method.“ In the “spit method,“ all subjects collected saliva in their mouths for 60 s and then spat it out into a graduated tube. The procedure was repeated for an additional 10 min. The subjects did not use any materials to stimulate secretion.

The samples were delivered to the Department of Medical Laboratory Diagnostics, Clinical Hospital Center Split, Split, Croatia, and then centrifuged at 1500× *g* for five minutes. The pad was then discarded into the infectious waste and the Salivettes were stored at −20 °C until used for study purposes. The frozen samples were first stored at room temperature for 30 min to determine the salivary cortisol concentration and salivary α-amylase activity. Then, the samples were centrifuged at 1500× *g* for five minutes. Salivary cortisol concentration was analyzed by the immunochemical method of enzyme-linked immunosorbent assays (ELISAs) using reagents from EUROIMMUN (Medizinische Labordiagnostika AG, Lübeck, Germany). The lower sensitivity limit of salivary cortisol concentration according to the manufacturer is 0.15 ng/mL; linearity ranges from 0.10 to 28.30 ng/mL. The coefficients of variation in the series were 3.70, 4.20, and 3.20 for the concentrations of 0.60, 2.10, and 13.40 ng/mL, and the coefficients of variation between series were 9.70, 7.90, and 4.70 for the concentrations of 1.30, 2.80, and 13.90 ng/mL. Saliva samples were analyzed using the Elysis Duo instrument (Human, Wiesbaden, Germany). Salivary α-amylase activity was measured by the kinetic colorimetric method using the Roche/Hitachi cobas c 701/702 Systems instrument with reagents from the same manufacturer.

After collection of UWS for the analysis of salivary cortisol concentration, serum samples were taken from the subjects to exclude hypercortisolemia due to other causes. Serum samples were collected one day after the collection of UWS to avoid the influence of the HPA axis on cortisol concentration during blood collection. The concentration of serum cortisol was measured by the immunochemical method of electrochemiluminescence (ECLIA) using the Roche/Hitachi cobas c e801 Systems instrument with reagents from the same manufacturer at the Department of Medical Laboratory Diagnostics, Clinical Hospital Center Split, Split, Croatia. The reference values for serum cortisol measured by this test are 171–536 nmol/L and refer to the morning test period.

The presence of blood in saliva (hemolysis) was tested by visual inspection, and saliva samples containing blood were excluded from the study [21,22].

### 2.4. Instruments

#### 2.4.1. Visual Analogue Scale (VAS)

The VAS (ranging from 0 to 100 mm) was used to assess the intensity of pain and/or burning (0 = no pain/burning, 100 = worst possible pain/burning) in patients with OLP and BMS.

#### 2.4.2. Depression, Anxiety and Stress Scale (DASS-21)

The psychological evaluation of each subject was assessed with the Depression, Anxiety, and Stress Scale (DASS-21, [23]). The same researcher collected the data through interviews to ensure that all subjects understood each question correctly. The scale consists of 21 statements and has a three-factor structure in the original, consisting of the subscales Depression, Anxiety, and Stress, each containing seven statements. They attempt to assess three negative emotional states, i.e., the degree of depression, anxiety, and stress in the past week. The reliability coefficients for the above subscales are 0.90 for the depression subscale, 0.89 for the anxiety subscale, and 0.91 for the stress subscale. The items are formulated as statements that are rated on a four-point Likert scale. The scale ranges from 0—does not apply to me at all, to 3—applies to me almost completely or most of the time. The score for the subscales was calculated by adding the scores for the seven statements and multiplying them by the number two. The maximum score for each subscale is 42, i.e., a higher score for each subscale means a higher level of depression, anxiety, or stress [24].

### 2.5. Statistical Analysis

All statistical analyses were performed with MedCalc software (MedCalc Software, Ostend, Belgium, version 22.007). All graphical figures were made using SigmaPlot for Windows^®^ software (Systat Software Inc., San Jose, CA, USA, version 14.0). Quantitative data are presented as the mean ± standard deviation or median and interquartile range (IQR), while qualitative data are presented as a whole number and percentage. Normality of the distribution was estimated using the Kolmogorov–Smirnov test. Student’s *t*-test for independent samples and Mann–Whitney U test were used for comparisons of quantitative variables between two groups. The chi-square (χ^2^) test was used for comparisons of qualitative variables between groups. The correlations were estimated using Spearman correlation. The comparison of quantitative variables between three groups was performed using one-way ANOVA with the Scheffe’s *post hoc* test or the Kruskal–Wallis test with Dunn’s *post hoc* test. The level of statistical significance was set at *p* < 0.05.

## 3. Results

### 3.1. Study Subjects

The sample of OLP patients consisted of 60 subjects ranging in age from 22 to 86 years, median 63.0 (IQR 51.5 to 70.5). The sample of BMS patients consisted of 60 subjects aged 27 to 86 years, median 66.0 (IQR 57.0 to 72.0). The control group consisted of 40 subjects aged 41 to 80 years, median 61.0 (IQR 52.0 to 65.0). In all three groups, the majority of subjects were women (75.0% vs. 81.7% vs. 82.5%). There was no statistically significant difference between the groups in regard to age and gender (*p* = 0.10, *p* = 0.57, respectively) (Table 1).

The mean disease duration was 18.5 months (9.0–36.5) in patients with OLP, while it was 15.0 months (8.0–36.0) in patients with BMS (*p* = 0.76, Mann–Whitney U test). The patients with primary BMS had statistically significant higher VAS scores compared to patients with OLP (7.0 vs. 3.5) (*p* < 0.001, Mann–Whitney U test).

### 3.2. Salivary Biomarkers

**Figure 1:**
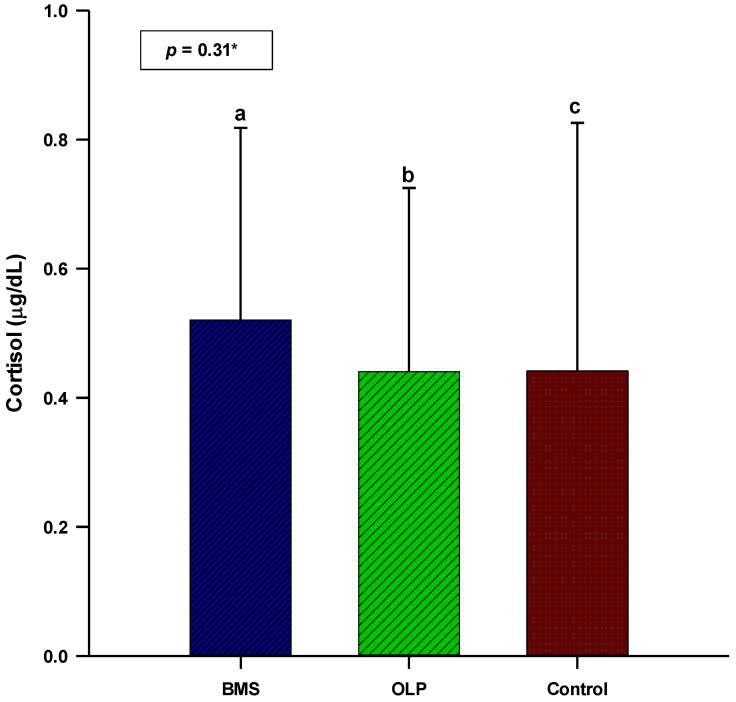
Comparison of salivary cortisol concentration between patients with OLP (N = 60), patients with primary BMS (N = 60), and control subjects (N = 40). * One-way analysis of variance (ANOVA) with *post hoc* Scheffe test. ^a^ BMS: 0.52 ± 0.29; ^b^ OLP: 0.44 ± 0.28; ^c^ control: 0.44 ± 0.38. Abbreviations: BMS, burning mouth syndrome; OLP, oral lichen planus.

**Figure 2:**
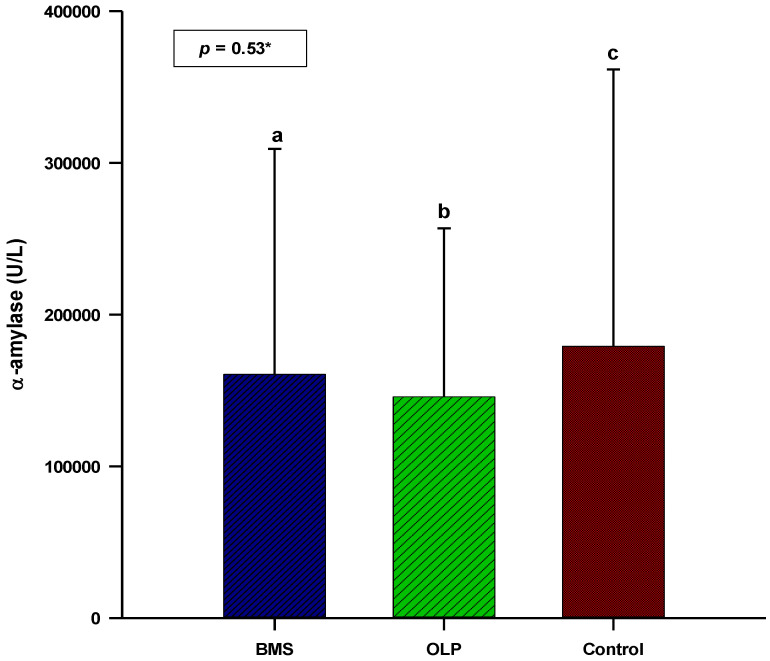
Comparison of salivary α-amylase activity between patients with OLP (N = 60), patients with primary BMS (N = 60), and control subjects (N = 40). * One-way analysis of variance (ANOVA) with *post hoc* Scheffe test. ^a^ BMS: 160,531 ± 148,806; ^b^ OLP: 145,804 ± 111,087; ^c^ control: 179,107 ± 182,639. Abbreviations: BMS, burning mouth syndrome; OLP, oral lichen planus.

The patients with primary BMS had higher salivary cortisol concentrations and α-amylase activity (0.52 vs. 0.44 µg/dL; 160,531 vs. 145,804 U/L) compared to patients with OLP. The concentration/activity of salivary cortisol and α-amylase showed no statistically significant difference between patients with OLP (N = 60) or primary BMS (N = 60) and control subjects (N = 40) (*p* = 0.31; *p* = 0.54) (Figure 1 and Figure 2).

### 3.3. Psychological Profile

The patients with primary BMS had statistically significant higher scores for depression, anxiety, and stress compared with patients with OLP and control subjects (*p* < 0.001, Kruskal–Wallis test) (Table 2). Stress was the leading mental disorder in patients with OLP and primary BMS, and stress scores were twice as high in patients with primary BMS as in patients with OLP. The patients with primary BMS had ten times higher depression scores compared to patients with OLP.

There was no correlation between the concentration/activity of salivary biomarkers (cortisol, α-amylase) and the psychological profile of patients with OLP and patients with primary BMS. There was a strong positive correlation between anxiety and depression (r = 0.643, *p* < 0.001), stress and depression (r = 0.720, *p* < 0.001), and stress and anxiety (r = 0.696, *p* < 0.001) in patients with OLP (Table 3). There was a strong positive correlation between anxiety and depression (r = 0.652, *p* < 0.001), stress and depression (r = 0.793, *p* < 0.001), and stress and anxiety (r = 0.705, *p* < 0.001) in patients with primary BMS (Table 3).

There was a positive correlation between disease duration and salivary cortisol concentration in patients with OLP (r = 0.253, *p* = 0.05). There was no correlation between disease duration and psychological profile in patients with OLP. There was no correlation between disease duration and concentration/activity of salivary biomarkers (cortisol, α-amylase) and psychological profile in patients with primary BMS (Table 4).

There was no correlation between symptom intensity (pain/burning) and concentration/activity of salivary biomarkers (cortisol, α-amylase) and psychological profile in patients with OLP. There was no correlation between symptom intensity (pain/burning) and the concentration/activity of salivary biomarkers (cortisol, α-amylase) in patients with primary BMS. There was a good positive correlation between symptom intensity (pain/burning) and psychological profile (depression, anxiety, stress) in patients with primary BMS (r = 0.373, *p* = 0.003; r = 0.515, *p* < 0.001; r = 0.365, *p* = 0.004, respectively) (Table 4).

### 3.4. Erosive and Non-Erosive Forms of OLP

There was no difference in the concentrations/activity of salivary stress biomarkers (cortisol, α-amylase) and the psychological profile of patients with erosive and non-erosive forms of OLP (Table 5).

## 4. Discussion

OLP and primary BMS are two oral diseases whose different etiopathogenetic mechanisms are complex, i.e., psychoneuroimmunoendocrinological, and not fully elucidated. Therefore, in this study, we observed the concentration/activity of salivary stress biomarkers (cortisol, α-amylase) and the psychological profile of patients with OLP, patients with primary BMS, and control subjects.

Salivary cortisol concentration showed no statistically significant difference between patients with OLP, patients with primary BMS, and control subjects (*p* = 0.31). The results of the different studies on salivary cortisol concentration in patients with OLP are contradictory. The meta-analysis by Lopez-Jornet P et al. analyzed salivary cortisol concentrations in patients with OLP in six studies. All studies were of high quality (according to the Newcastle–Ottawa quality assessment scale). This meta-analysis showed a statistically significant difference in salivary cortisol concentrations (by ELISA) in the population of Indian patients with OLP compared to control subjects (5.33 ng/mL, *p* < 0.0001), but not in studies conducted in the Middle East and Europe [25]. A systematic review by Humberto JSM et al. included five studies that analyzed salivary cortisol concentrations (by ELISA) in patients with OLP [26]. Three of them showed increased salivary cortisol concentrations in patients with OLP compared to control subjects [27,28,29]. Most studies in this systematic review had a low level of evidence and a weak level of recommendation (level 3b/grade B) [26]. Rödström PO et al., Girardi C et al., Nosratzehi T et al., and Pippi R et al. found no difference in salivary cortisol concentrations between patients with OLP and control subjects [30,31,32,33]. Their findings are consistent with the results of our study. The absence of a difference in salivary cortisol concentrations between patients with OLP and control subjects in our study may be explained by ethnicity (European), different waking times of subjects, different timing of sample collection, and different diagnostic inclusion criteria for OLP patients. We made an attempt to reduce bias by only comparing studies in which the salivary cortisol concentrations were determined by ELISA.

In our study, there was no statistically significant difference in salivary cortisol concentrations between patients with erosive and non-erosive forms of OLP (0.45 ± 0.31 vs. 0.41 ± 0.23 µg/dL, *p* = 0.69). The study included twice as many patients with the erosive form of OLP compared to the non-erosive form. Our results are consistent with those of Lopez-Jornet P et al. and Mansourian A et al. who found no statistically significant difference in salivary cortisol concentrations between the different clinical forms of OLP [34,35].

The etiology of primary BMS is multifactorial, and alteration of cortisol concentrations is thought to play a possible role [36]. The results of published studies are contradictory. A systematic review by Aitken-Saavedra J et al. showed increased salivary cortisol concentrations in patients with primary BMS [37]. The systematic review and meta-analysis by Fernández-Agra M et al. included studies with low heterogeneity that analyzed 54 different biomarkers in patients with BMS. However, salivary cortisol was the only biomarker included in the meta-analysis and the results showed a statistically significant higher salivary cortisol concentration in patients with BMS compared to control subjects (mean 0.39, *p* = 0.003) [20]. López-Jornet P et al., Nakagawa A et al., Nosratzehi T et al., and de Souza FTA et al. showed no statistically significant difference in salivary cortisol concentration between patients with primary BMS and control subjects [14,38,39,40]. Their results are consistent with the results of our study. The differences in the obtained results could be due to the different waking times of the subjects (which we could not influence), different timing of sample collection, and different diagnostic inclusion criteria for BMS. The experts of the working group of the World Workshop in Oral Medicine VII pointed out the great heterogeneity of the definition and diagnostic criteria for BMS in clinical trials [41].

The absence of a statistically significant difference in salivary cortisol concentrations between the three groups of subjects (patients with OLP, patients with primary BMS, control subjects) indicates impaired regulation of the HPA axis, i.e., its hypoactivity in these chronic diseases and painful conditions. The salivary cortisol concentration was higher in patients with BMS than in patients with OLP. In addition, there was a positive correlation between disease duration and salivary cortisol concentration in patients with OLP. This suggests a gradual recovery of the HPA axis and better adaptive coping mechanisms in stressful situations in patients with OLP compared with patients with BMS.

Salivary α-amylase activity showed no statistically significant difference between patients with OLP, patients with primary BMS, and control subjects (*p* = 0.54). There are few studies that have investigated salivary α-amylase activity in patients with OLP, and their results are contradictory. The results of the study by Simour JAdS et al. showed increased salivary α-amylase activity 30 min after awakening in patients with OLP (determined at three time points) [42]. Pippi R et al. showed no statistically significant difference in daily fluctuation or production of salivary α-amylase between patients with OLP and control subjects (determined at three time points) [14]. Their results are consistent with the results of our study. Moreover, there was no statistically significant difference in salivary α-amylase activity between patients with erosive and non-erosive forms of OLP in our study (100,815 vs. 101,035 U/L, *p* = 0.55).

Most of the studies performed showed the association between salivary α-amylase and BMS. The systematic review by Aitken-Saavedra J et al. and the systematic review and meta-analysis by Fernández-Agra M et al. showed increased salivary α-amylase activity in patients with primary BMS [20,37]. Kim H-I et al. showed no difference in salivary α-amylase activity between patients with BMS and control subjects [43]. Their results are consistent with the results of our study. Discrepancies in the results of salivary α-amylase activity can be explained by the different wake-up times of the subjects, the different times for sample collection, and the different diagnostic inclusion criteria for OLP and BMS. The objective relationship between chronic stress and salivary α-amylase activity is still the subject of numerous studies. Because BMS is a chronic pain disorder, we did not determine this association in our study. Salivary α-amylase activity was higher in patients with primary BMS compared with patients with OLP. This suggests that salivary α-amylase may be a better indicator of stress than salivary cortisol, which was also shown in the study by Nosratzehi T et al. [14].

The patients with primary BMS had a worse psychological profile, i.e., higher scores for depression, anxiety, and stress compared with patients with OLP and control subjects (*p* < 0.001). The results of the studies conducted on the association between psychological disorders and OLP are contradictory. Simoura JAdS et al. showed a significant correlation between depression, anxiety, and stress, and OLP [42]. The systematic review and meta-analysis by De Porras-Carrique T et al. showed a strong association between OLP and mental disorders. It is important to note that this meta-analysis was of low methodological quality (according to AMSTAR2) [44]. Girardi C et al. did not find statistically significant higher stress scores between patients with OLP and control subjects [31]. Nevertheless, numerous studies support the association between OLP and mental disorders. This was also evident in our study, as anxiety and stress scores were twice as high in patients with OLP as in control subjects. There was no statistically significant difference in depression, anxiety, and stress between patients with erosive and non-erosive forms of OLP (1.0 vs. 2.0, 3.0 vs. 5.0, 8.0 vs. 8.0, *p* = 0.51, *p* = 0.54, *p* = 0.76, respectively). Our results are consistent with those of Shah B et al. and Ebrahimi H et al. [28,45].

Numerous studies have demonstrated a strong association between mental disorders and BMS. This was also confirmed by the results of our study. López-Jornet P et al., Koike K et al., and de Pedro M et al. showed an association between depression, anxiety, and stress and BMS [38,46,47]. Comparing the psychological profile of patients with OLP and primary BMS, we can conclude that the statistically significant higher scores of depression, anxiety, and stress in patients with primary BMS indicate a strong association between psychogenic factors and BMS. The patients with BMS had a ten-fold higher depression score and two-fold higher stress scores compared with patients with OLP. Psychogenic factors are part of the multifactorial etiology of BMS. The discrepancies in the psychological profiles of patients with OLP and BMS can be explained by the use of different diagnostic tools to identify mental disorders and by the cultural differences of the subjects.

The results of our study suggest that depression, anxiety, and stress are a consequence of OLP rather than a cause (although this cannot be determined with this type of study). Disease duration showed no correlation with the psychological profile of patients with OLP and BMS. This indicates good adaptation mechanisms of these patients to chronic pain diseases/conditions. The results of our study showed no correlation between symptom intensity (pain/burning) and psychological profile in patients with OLP. In contrast, patients with primary BMS showed a good positive correlation between symptom intensity (pain/burning) and psychological profile, indicating a close relationship and a possible causal role. The DASS-21 could be an initial and useful tool for early detection of psychological disorders. Psychiatrists should be involved in diagnosing comorbidity in patients with OLP and primary BMS.

The advantage of our study is the sample size. Our case–control study is the first to compare the concentration/activity of salivary biomarkers in two different oral psychoneuroimmunoendocrine diseases/disorders. In addition, our study assessed oral hygiene, which has only been performed in a single study or was not reported in the available literature [48]. Indeed, it is known that poor oral hygiene can increase the concentration/activity of some salivary biomarkers [49].

Our case–control study has several limitations. We determined the concentration/activity of salivary biomarkers at a single time point. In addition, the concentration/activity of salivary biomarkers was not corrected using the concentration of total proteins. It is recommended that more sensitive methods (determination of hemoglobin, transferrin) be used to detect blood in saliva rather than visual inspection. It is known that some drugs can have a long-term effect of more than three months, which is a potential confounding factor [50]. Another confounding factors is the varying sensitivity of the kits.

Future studies should be controlled longitudinal clinical studies with strict inclusion criteria: use of UWS as a reference for salivary biomarker determination, standardization of methods for salivary biomarker determination, ethnicity of subjects (stratification), use of agreed international diagnostic criteria for OLP and BMS, attention to the prevalence of different clinical forms of OLP, use of an international measure for assessing salivary α-amylase activity, and assessment of oral hygiene.

## 5. Conclusions

The patients with primary BMS had higher salivary cortisol concentrations and α-amylase activity compared with patients with OLP. In addition, patients with primary BMS had ten times higher depression scores and twice as high anxiety and stress scores, i.e., poorer mental health, compared with patients with OLP. There was a strong positive correlation between anxiety and depression, stress and depression, and stress and anxiety in patients with OLP and BMS. This suggests the importance of cortisol and salivary α-amylase as stress biomarkers, considering the demonstrated strong association between BMS and mental disorders. An interdisciplinary psychoneuroimmunological approach is necessary for chronic diseases/disorders such as OLP and BMS because they are related to the patient’s psychological state, regardless of whether mental disorders are a cause or a consequence.

## Figures and Tables

**Table 1:** Demographic data.

	Sample N = 160	OLP Group N = 60	BMS Group N = 60	Control Group N = 40	*p*
Gender (N, %) Men Women	33 (20.6) 127 (79.4)	15 (25.0) 45 (75.0)	11 (18.3) 49 (81.7)	7 (17.5) 33 (82.5)	0.57 *
Age (years)	63.0 (52.0–70.0)	63.0 (51.5–70.5)	66.0 (57.0–72.0)	61.0 (52.0–65.0)	0.10 ^#^

All data are presented as whole numbers (percentage) or median (interquartile range). * Chi-square test. ^#^ Kruskal–Wallis test with *post hoc* Dunn test. Abbreviations: BMS, burning mouth syndrome; OLP, oral lichen planus.

**Table 2:** Psychological profile of patients with OLP, primary BMS, and control subjects.

	OLP Group N = 60	BMS Group N = 60	Control Group N = 40	*p* *
Depression	1.0 (0.0–8.0)	10.0 (4.0–18.0)	2.0 (0.0–4.0)	<0.001 ^ab^
Anxiety	4.0 (0.0–8.0)	7.0 (4.0–16.0)	2.0 (0.0–5.0)	<0.001 ^ab^
Stress	8.0 (2.0–14.0)	16.0 (8.0–28.0)	4.0 (2.0–14.0)	<0.001 ^ab^

* Kruskal–Wallis test with *post hoc* Dunn’s test. ^a^ BMS vs. OLP; *p* < 0.05. ^b^ BMS vs. control; *p* < 0.05. OLP vs. control; *p* < 0.05. Abbreviations: BMS, burning mouth syndrome; OLP, oral lichen planus.

**Table 3:** Correlation of concentration/activity of salivary biomarkers (cortisol, α-amylase) and psychological profile in patients with OLP (N = 60) and primary BMS (N = 60).

		Depression	Anxiety	Stress	Cortisol	α-Amylase
OLP	Depression	1.0				
	Anxiety	0.643 *	1.0			
	Stress	0.720 *	0.696 *	1.0		
	Cortisol	−0.011	−0.016	−0.077	1.0	
	α-Amylase	−0.037	0.050	0.053	0.039	1.0
BMS	Depression	1.0				
	Anxiety	0.652 *	1.0			
	Stress	0.793 *	0.705 *	1.0		
	Cortisol	0.083	0.028	0.122	1.0	
	α-Amylase	0.048	−0.218	−0.024	0.008	1.0

* *p* < 0.001; Spearman’s correlation.

**Table 4:** Correlation of disease duration and symptom intensity (pain/burning) with concentration/activity of salivary biomarkers (cortisol, α-amylase) and psychological profile in patients with OLP (N = 60) and primary BMS (N = 60).

		OLP		BMS	
		r *	*p*	r *	*p*
Disease duration	Cortisol	0.253	0.05	−0.089	0.50
	α-Amylase	0.038	0.77	0.076	0.56
	Depression	−0.078	0.55	−0.007	0.96
	Anxiety	−0.035	0.79	−0.033	0.80
	Stress	−0.023	0.86	−0.026	0.84
Symptom intensity (pain/burning)	Cortisol	0.006	0.96	−0.230	0.08
	α-Amylase	0.079	0.55	−0.004	1.10
	Depression	0.109	0.41	0.373	0.003
	Anxiety	0.081	0.54	0.515	<0.001
	Stress	0.089	0.50	0.365	0.004

* Spearman’s correlation coefficient. Abbreviations: BMS, burning mouth syndrome; OLP, oral lichen planus.

**Table 5:** Comparison of concentration/activity of salivary biomarkers (cortisol, α-amylase) and psychological profile between patients with erosive and non-erosive forms of OLP.

	Erosive OLP	Non-Erosive OLP	
	N = 40	N = 20	*p*
Cortisol	0.45 ± 0.31	0.41 ± 0.23	0.69 ^†^
α-Amylase	100,815 (67,160–257,030)	101,035 (74,825–142,870)	0.55 *
Depression	1.0 (0.0–5.0)	2.0 (0.0–10.0)	0.51 *
Anxiety	3.0 (0.0–6.0)	5.0 (0.0–13.0)	0.54 *
Stress	8.0 (2.0–12.0)	8.0 (1.0–16.0)	0.76 *

* Mann–Whitney U test; ^†^ independent *t*-test. Abbreviation: OLP, oral lichen planus.

## Data Availability

Data are available upon request through the corresponding author’s e-mail.
